# Supplementary material for: Impact of Genetic Variations on Thromboembolic Risk in Saudis with Sickle Cell Disease
Source: Genes (Basel). 2023 Oct 9;14(10):1919. doi: 10.3390/genes14101919 (PMC10606407; doi:10.3390/genes14101919)
Supplement: Supplementary file 1 [file genes-14-01919-s001.zip › genes-2535347-supplementary Figure S1.pdf]

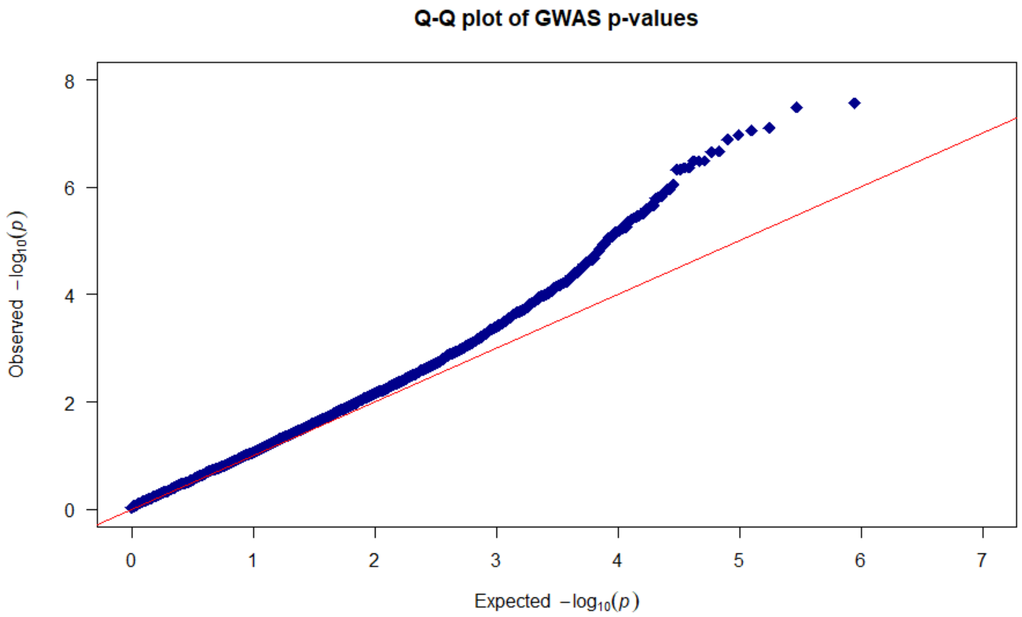

Supplementary Figure S1: GWAS results plot of SCD cohort (65 TEE cases vs 285 controls). Q–Q plot of the observed and expected P-values generated from an allelic genetic model which involved a set of 683,030 variants. The expected values plot is shown in red in comparison to the observed values plot in blue.
